# Bilateral Ewing Sarcoma/Primitive Neuroectodermal Tumor of the Breast: A Very Rare Entity and Review of the Literature

**DOI:** 10.1155/2013/964568

**Published:** 2013-05-30

**Authors:** N. Majid, M. Amrani, I. Ghissassi, M. El Cadi, M. El Bouzidi, M. El Kabous, A. Kherbach, H. Errihani

**Affiliations:** ^1^Department of Medical Oncology, National Institute of Oncology, Rabat, Morocco; ^2^Department of Pathology, National Institute of Oncology, Rabat, Morocco; ^3^Department of Gynecology and Obstetrics, Faculty of Medicine and Pharmacy, University Mohammed V Souissi, Rabat, Morocco

## Abstract

Peripheral primitive neuroectodermal tumors (PNET) are rare malignant tumors, affecting mostly children and adolescents and have been described in breast in eight case reports only. In this paper, we present a case of bilateral mammary ES/PNET where distinction between primary and metastatic diseases was discussed through a literature review. The aim of this work is to demonstrate that although rare, the possibility of PNET should be kept in mind while evaluating a palpable breast abnormality in a young female.

## 1. Introduction 

The ES/PNET family of tumors is part of a rare group of malignant neoplasms arising from neuroectodermal elements, with small round cell morphology. This variant typically occurs in bony structures of adolescents and young adults [1]. The diagnosis of ES/PNET requires immunohistochemistry analysis and the presence of a t(11;22) translocation. As a soft tissue neoplasm, PNET arising in the breast is extremely uncommon; only 8 cases were reported in an extensive search in the medical literature. To our knowledge, none had bilateral breast involvement as presented in this case.

## 2. Case Report

**Figure 1:**
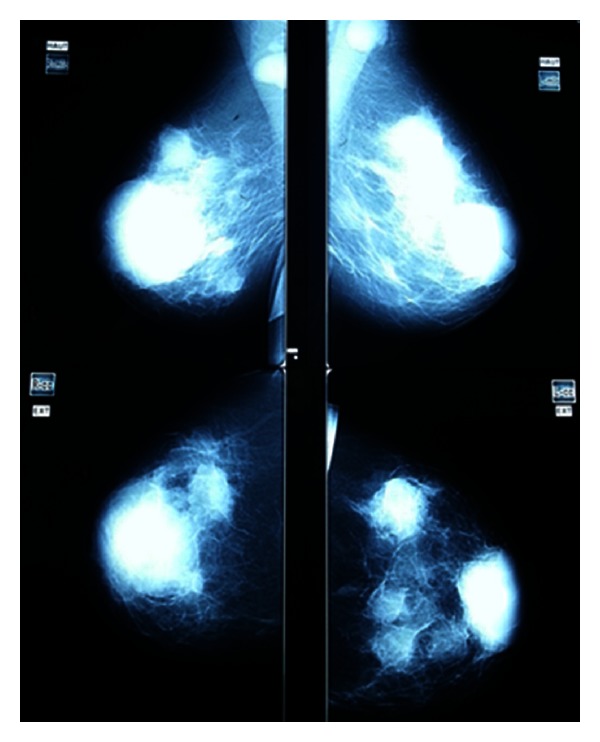
The mediolateral oblique (MLO) and craniocaudal (CC) view of the left and right breast mammogram.

**Figure 2:**
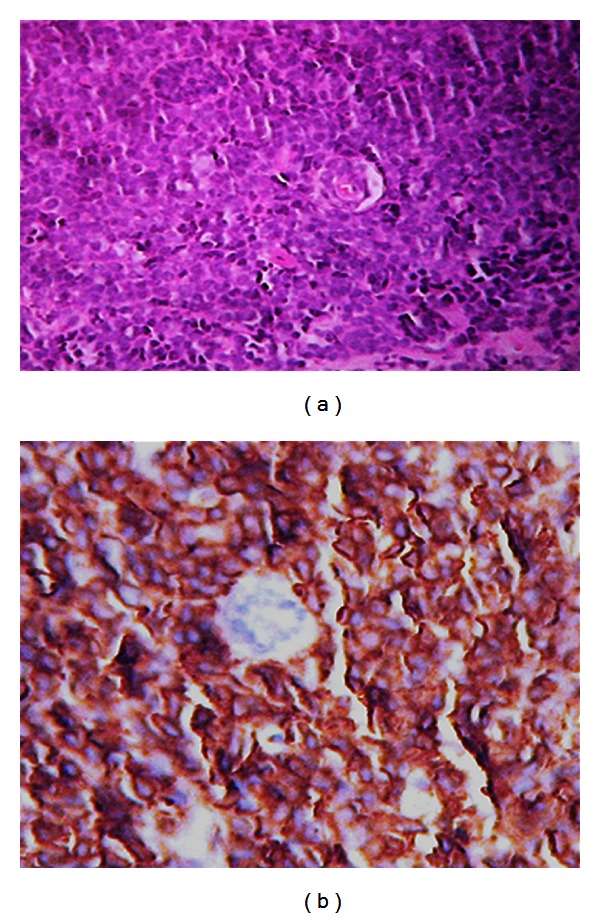
Sheets of small round cells. Hematein-eosin stain ×40 (a); CD99 membranous staining of tumor cells ×40 (b).

A 30-year-old woman presented with painless and progressively growing lumps in the right than in the left breast for 10 months duration. There was no family history of breast cancer, prior breast mass, trauma, or other associated symptoms. Examination revealed firm, fixed, painless, and palpable retromammary bilateral masses measuring 7 and 4 cm in the right and left breast, respectively, associated with skin retraction and bilateral axillary lymph node metastases. Mammography and ultrasonography identified suspicious multiple bilateral masses. The lesions were hypoechoic, heterogeneous with skin thickening predominant in the periareolar area without evidence of microcalcifications (Figure 1); axillary lymph nodes were enlarged. The pathology report of bilateral biopsies performed showed a proliferation of small, round to oval cells having unconspicuous nucleoli and scanty cytoplasm with thickened nuclear membrane (Figure 2(a)). Tumor cells were strongly positive for vimentin and CD99 (Figure 2(b)) but were negative for AE1/AE3, leukocyte common antigen LCA, chromogranin, and CD56. Disease progressed rapidly and the patient became symptomatic with considerable dyspnea. A staging workup with whole body computed tomography scan and bone scintigraphy revealed a superior mediastinal mass extending to the para-aortic area with metastases in the right lung and a pleural effusion; no bone metastasis was found. Based on these findings metastatic ES/PNET was the final diagnosis. Therefore the patient received 2 cycles of VAC IE regimen (cyclophosphamide 1200 mg/m^2^ iv d1 followed by mesna, doxorubicin 75 mg/m^2^ iv bolus d1, vincristine 2 mg iv d1 alternating with Ifosfamide 1.8 g/m^2^/d iv d1–5 given with mesna, Etoposide 100 mg/m^2^/d iv d1–5 every 21 days), but unfortunately she succumbed to respiratory failure due to pulmonary metastasis and she died. 

## 3. Discussion

 Ewing's sarcoma (EWS)/peripheral primitive neuroectodermal tumors (PNET) are small round cell tumors, occurring primarily in bone and soft tissues of the limbs [1] and arise from neuroectodermal elements that probably develop from migrating embryonic cells of the neural crest [10].

This group of tumors is characterized by the presence of the typical translocation (11;22) (q24;q12) and the expression of CD99 antigen (MIC2) on immunohistochemistry [11], as seen in this case. Children and young adults are most frequently affected, and our patient was 30 years of age. As soft tissue neoplasms, PNET/ES have been described in the kidney, the parotid gland, the chest wall, the ovary, the rectum, the gall bladder, the retroperitoneal cavity, the myocardium, and the mediastinum [1]. Breast is an extremely rare location and has been reported only seven times as a primary tumor and one as a metastatic tumor, in a thorough search through the medical literature as described in Table 1.

 In the present case the distinction between primary and metastatic PNET to the breast was difficult. On one hand the clinical history suggested a primary PNET of the right breast which metastasized to the contralateral breast via lymph nodes localized along the anterior thoracic wall and then to the lung. Further, the most common metastatic tumors to the breast are from mammary primaries [12] in which case lymphatic metastases are usually found in the medial portion of the breast, the skin becomes diffusely thicker, and the breast parenchyma becomes denser on mammography with many irregular masses, which was found similar in our case. On the other hand, a primary mediastinal PNET that metastasized to the lung and the breast is also possible. In approximately 30% of patients, metastasis to the breast is the first sign of malignancy, and time from initial diagnosis to metastasis to the breast varies between 1month to 15years. Moreover, some reports emphasize that blood-born metastases to the breast are bilateral but often well-defined rounded masses in contrast to the present case [13]. Nevertheless, primary mediastinal PNET, even if uncommon, are mostly located in the posterior than in the anterior mediastinum like other neurogenic tumors [14].

Management of ES/PNET is usually multimodal, and patients with metastasis at diagnosis are treated with the same treatment approach as patients with localized disease, although prognosis is worse in the former group. The treatment comprises multidrug chemotherapy (vincristine, doxorubicin, cyclophosphamide, ifosfamide, and etoposide) and whole-lung irradiation in patients with lung metastases [15, 16]. In this case, the patient's medical condition deteriorated, and she died after 2 cycles of chemotherapy.

## 4. Conclusion 

 Prognosis of metastatic disease is generally poor and it does not seem to make a difference whether the ES/PNET is primary or metastatic to the breast. The objective of this case is to emphasize that histopathological confirmation is mandatory especially in cases of unusual locations.

## Figures and Tables

**Table 1:** Summary of primitive neuroectodermal tumors of the breast reported in the literature.

Reference	Age (years)	Presentation	Size (cm)	Disease	Treatment	Outcome
Tamura et al. [2]	47	Breast lump	2.1×1.8	Primary	Mastectomy	Not available

Maxwell et al. [3]	35	Breast lump	1.8	Primary	Lumpectomy + chemotherapy	Free of disease at 2.5 years

da Silva et al. [4]	35	Breast lump	12 ×7.5	Primary	Chemotherapy + radiotherapy	Local and pulmonary relapse; death at 2 years

Ko et al. [5]	33	Breast lump	2.5× 2	Primary	Lumpectomy	Free of disease at 6 months

Vindal and Kakar [6]	26	Breast lump	3 × 2	Primary	Wide local excision + adjuvant chemotherapy	Free of disease at 36 months

Kwak et al. [7]	49	Mass in the axilla		Metastatic	Chemotherapy	Not available

Dhingra et al. [8]	26	Breast lump	3.5× 3	Primary	Mastectomy + chemotherapy + radiotherapy	Free of disease at 12 months

Suebwong et al. [9]	46	Breast lump	4	Primary	Chemotherapy + radiotherapy	Local and pulmonary progression

Majid et al.	30	Bilateral breast lump	7 and 5 in the right and left, respectively	Metastatic	Chemotherapy	The patient's medical condition deteriorated, and she died after 2 cycles of chemotherapy
